# Association between adherence to Saudi healthy plate dietary guidelines and physical activity, muscular endurance, and body composition among adult gym users

**DOI:** 10.3389/fnut.2026.1940147

**Published:** 2026-09-16

**Authors:** Asrar Suruji, Areej Alkhaldy, Wejdan Alghafari, Dina Qahwaji

**Affiliations:** Department of Clinical Nutrition, Faculty of Applied Medical Sciences, King Abdulaziz University, Jeddah, Saudi Arabia

**Keywords:** body composition, dietary adherence, dietary guidelines, gym users, muscular endurance, physical activity

## Abstract

**Introduction:**

Many gym users prioritize nutritional supplementation and dietary restriction over following the dietary guidelines that is necessary for optimal health to promote physical performance. This study aimed to assess the adherence of male and female gym attendees in Makkah city, Saudi Arabia, to the Saudi Healthy Plate Dietary Guidelines (SHPDGs) in relation to physical activity, muscular endurance, and body composition.

**Methods:**

This study is cross-sectional design involved 268 regular users of Makkah gyms aged 18 years or older (129 males [48.1%] and 139 females [51.9%]). An electronical questionnaire was used from November 2024 to April 2025 to collect information on demographics, adherence to the SHPDGs, and physical activity levels. Muscular endurance was assessed via a standardized push-up test. Body composition was measured using bioelectrical impedance analysis (InBody).

**Results:**

The study found that 12.7% of participants had low adherence to the SHPDGs, 74.3% of participants had moderate adherence, and 13.1% of participants had high adherence. Males showed significantly greater adherence to healthy dietary patterns, higher physical activity levels, and higher muscular endurance than females (*p* < 0.001). SHPDG adherence was associated with a significant reduction in fat mass (*p* = 0.007) and increases in muscle mass (*p* < 0.001) and total body water (*p* = 0.022). Higher adherence to the SHPDGs enhanced physical activity and upper-body endurance (*p* < 0.001 and *p* = 0.029, respectively).

**Conclusion:**

Adherence to the SHPDGs was linked to improved physical activity levels, body composition, and muscular endurance. These findings emphasize the importance of promoting balanced nutrition among gym users to support physical performance and overall health.

## 1 Introduction

Dietary patterns and physical activity are closely interrelated, and their synergistic effects have been extensively studied. Both are recognized as primary modifiable factors in reducing the risk of non-communicable diseases (1–3). Beyond disease prevention, they support musculoskeletal function (4), body composition (5, 6), cognitive function (7), and overall wellbeing (8). In this context, optimal nutrition and adherence to dietary guidelines play essential roles in promoting long-term healthy habits and providing physically active individuals with the essential macro- and micronutrients that are required for normal physiological functions (9, 10).

Multiple studies have explored the positive relationship between adherence to dietary guidelines and physical fitness (11–13), muscular fitness (11–13), and body composition (6, 11–14). A meta-analysis conducted by Bizzozero-Peroni et al., analyzing data from over 36,000 adults, revealed that high adherence to a Mediterranean diet was significantly associated with improved cardiorespiratory, musculoskeletal, and overall physical fitness (15). In addition, García-Hermoso et al. reported positive associations between Mediterranean diet adherence and both cardiorespiratory fitness and muscular fitness in a systematic review and meta-analysis involving more than 565,000 young people (16). Moreover, adherence to the 2010 Dietary Guidelines for Americans was inversely associated with adiposity in young women, with those in the highest adherence quartile exhibiting significantly lower percent body fat (17), while Mogna-Peláez et al. reported that greater adherence to the Planetary Health Diet during energy restriction led to reductions in body weight, body mass index (BMI), and fat mass (18).

Gym users represent a nutritionally distinct subgroup; their nutritional needs must satisfy both standard dietary guidelines and the additional demands of regular training and body composition goals. Gym users who fail to achieve sufficient energy intake and lack a well-balanced macronutrient profile may encounter significant complications in their training adaptation and recovery, including reduction of fat-free mass, compromised immune function, diminished bone mineral density, heightened injury susceptibility, and more severe overtraining syndrome symptoms (19). Hence, the adequacy of nutrition plays a fundamental role. For instance, the consumption of adequate carbohydrate promotes glycogen replenishment and sustains muscular output during repeated effort, while sufficient protein intake supports skeletal muscle hypertrophy, repair, and functional preservation (20, 21). Furthermore, the micronutrients intake, including vitamins C and D, calcium, and iron, supports neuromuscular function and the processes of recovery to enhance endurance (21–25). However, few studies have examined whether adherence to country-specific dietary guidelines is associated with differences in muscular endurance performance or body composition (11, 26–28).

In Saudi Arabia, the fitness market has undergone a remarkable expansion due to investment in wellness infrastructure (29). This is linked to the Quality of Life Programme (QoLP), which was launched in 2018 as a key pillar of the national Vision 2030 strategy with the goal of realizing a population that is more physically active, better nourished, living longer, and less burdened by preventable chronic diseases (30). In addition, to ensure nutritional adequacy and reduce the burden of non-communicable diseases, the Saudi Healthy Plate Dietary Guidelines (SHPDGs) were developed (31). Despite the development of these guidelines, no published studies have assessed SHPDG adherence in physically active populations or gym users, nor have any examined the relationship between adherence and physical fitness outcomes such as muscular endurance and body composition. Furthermore, most of the existing studies among gym users focused on the prevalence dietary supplement (32), use of protein supplements (33), alongside anabolic-androgenic steroid use (34), with only one study examining the nutritional knowledge and dietary intake of gym practitioners and athletes in Riyadh (35). The study found that nutritional knowledge was poor, with low fruit, vegetable, and dairy intake and high meat and protein intake (35). These findings are not following the recommended dietary practices and show a gap in the adherence of dietary guideline among physically active individuals, which may limit the understanding of how well these individuals meet the dietary recommendations.

Many gym users are goal-oriented, with common aims being to lose weight, build muscle, or improve overall fitness. Thus, they may adhere more to dietary restrictions and nutritional supplementation than to the SHPDGs. Despite the well-established impact of dietary quality on active populations, no studies have specifically examined adherence to the SHPDGs among gym users in Saudi Arabia and its relationship to functional fitness and body composition. Therefore, the aim of this study was to assess SHPDG adherence in relation to three outcomes: (1) physical activity, (2) muscular endurance, and (3) body composition among male and female Saudi gym users. We hypothesized that higher adherence to SHPDGs would be positively associated with physical activity level, muscular endurance, and body composition among Saudi gym users.

## 2 Materials and methods

### 2.1 Study design and participants

This cross-sectional study was conducted between November 2024 and April 2025. Ethical approval was obtained from the King Abdulaziz University Ethics and Research Committee with approval number 351–24. All participants gave their consent to participate and were provided with an explanation of the study purpose, the study requirements, and their right to withdraw. The participants were male and female Saudi adults aged between 18 and 40 years who had been physically active for a minimum of 3 months. The participants were recruited from local gyms in Makkah city. The exclusion criteria included individuals with chronic disease that might affect physical activity performance or dietary intake (e.g., diabetes mellitus, cardiovascular disease, chronic kidney disease, and thyroid or other endocrine disorders). In addition, gym users reported current use of performance enhancing drugs or hormonal supplements were also excluded. A total of 333 gym users were initially assessed for eligibility. Of these, 65 were excluded due to non-adherence to regular gym activity (less than 3 months) and due to a chronic disease meeting the exclusion criteria. The final study sample comprised of 268 gym users.

### 2.2 Sample size

An online calculator (Epi Info online sample size calculator, developed by the Division of Health Informatics and Surveillance of the Center for Surveillance, Epidemiology, and Laboratory Services of the U.S. Centers for Disease Control and Prevention) was utilized (36). The required sample size was estimated based on the total number of 26,000 clients attending 14 popular gyms in Makkah. The required sample size for the current study was found to be *n* = 268 with a confidence level of 90% and a margin of error of 5%.

### 2.3 Study measures

An online self-reported questionnaire and objectively measured data were used to evaluate SHPDG adherence in relation to physical activity levels, muscular endurance, and body composition. The online self-reported questionnaire was constructed following an extensive literature review to assess sociodemographic data, dietary adherence to the SHPDGs, and physical activity level (31, 37, 38). The objectively collected data included anthropometric data, body composition, and an upper-body muscular endurance test.

#### 2.3.1 Study questionnaire

The first section of the questionnaire collected sociodemographic information including sex, education level, and marital status. The second section evaluated each respondent’s adherence to the SHPDGs as described by Al-Bisher and Al-Otaibi (37). This section evaluated daily intake of ten food groups, including fruits, vegetables, grains & bread, milk & dairy products, meat and meat substitutes, fats, fast food, soft drinks, crackers & crisps, and sweets & desserts. For each food group, participants can select the option that described their usual consumption (per day or per week) depending on the food group as shown in supplementary Table 1. Each option assigned a score range from 0 to 2 based on adherence to the dietary guidelines’ recommendation. For healthy food groups such as fruits, vegetables, protein foods, dairy products, and grains higher scores reflected lower or absent intake, while for unhealthy food groups such as those high in fat, sweets, crackers, fast foods, and soft drinks higher scores reflected more frequent intake. The scores of all 10 food groups were summed to range between 0 and 20, where a lower score indicated higher adherence to the SHPDGs. The total score for SHPDG adherence was classified into three categories: low adherence (14–20), moderate adherence (7–13), and high adherence (0–6).

The third section assessed the levels of physical activity using a translated version of the International Physical Activity Questionnaire (IPAQ) Short Form (38). This form includes questions about the time spent on activities and the intensity of activities in the last seven-consecutive-day period. Physical activity levels were divided into three categories: low, moderate, and high. Low physical activity refers to sedentary behavior or only walking. Moderate physical activity refers to at least 20 min of vigorous physical activity on three or more days, 30 min or more of moderate or low physical activity on five or more days, or 600 metabolic equivalent of task (MET) minutes/week by combining low, moderate, and vigorous activities. High physical activity refers to intense exercise on 3 days per week at 1,500 METs/min/week or at least 3,000 MET minutes/week through a combination of activities (38). Each activity category was assigned an estimated METs value: walking = 3.3 METs, moderate physical activity = 4 METs, and vigorous physical activity = 8 METs. The amount of time spent on each activity category was multiplied by their estimated value in METs to determine MET minutes/week: METs × minutes × days. Total METs was summed to gain an overall value of weekly physical activity (38).

#### 2.3.2 Muscular endurance levels (push-up test)

The push-up test was conducted in the presence of professionals trained to measure muscular endurance. All supervising professionals were instructed to use the same technique before and during the test. Male participants performed standard push-ups, while female participants had the option of performing either standard or modified push-ups. Before the actual test, the participants were given a 10 min warm up period standard push-ups by assuming a plank position, with the fingers pointing forward, arms shoulder-width apart, back straight, and head up, using the toes as the pivot point. Modified push-ups were performed in a plank position with the knees as the pivot point, lower legs in contact with the mat with the ankles plantar flexed. After that, participants elevated their body by fully straightening the elbows and returned to the down position, lowering their body until their elbows were at reach 90° and their chest was within 2 inches of the floor. This was done continuously without stopping until the participants could not follow the correct form. The number of push-ups performed was recorded and evaluated on the basis of normative data according to the participant’s age and sex, drawn from Golding et al. (39) (Supplementary Table 2).

#### 2.3.3 Anthropometric measurements

The participants’ weight, height, and body composition were measured. Height was measured in centimeters using a Harpenden stadiometer. Weight and body composition were measured using bioelectrical impedance analysis (InBody 270, Cerritos, CA, USA) (40). The InBody 270 analyzer employs a multifrequency eight-point tetrapolar touch electrode system to accurately measure body impedance across different body segments and provide an analysis of body fat percentage and muscle mass. Body mass index (BMI) was calculated by dividing body weight in kilograms by the square of body height in meters. The BMI values were classified according to the Centers for Disease Control and Prevention (CDC) into underweight (≤18.5 kg/m^2^), normal (18.5–24.9 kg/m^2^), overweight (25–29.9 kg/m^2^), obesity class I (30–34.9 kg/m^2^), obesity class II (35–39.9 kg/m^2^), and obesity class III (≥40 kg/m^2^) (41). The examination took 1 min, during which time participants were instructed to stand barefoot with their soles in contact with the foot electrodes and their palms and thumbs in contact with the electrodes on the handles with their arms abducted. In addition, participants were instructed to avoid exercising for 6 to 12 h, eating or drinking for at least 3 to 4 h, taking a shower or sauna, or putting lotion or ointment on their hands or feet prior to measurement. They were also asked to empty their bladders and remove all shoes, pantyhose, socks, heavier clothing, and metal items such as belts, watches, and jewelry (40). The analysis parameters included body weight, BMI, obesity degree, protein mass, muscle mass, fat mass, body fat percentage, and total body water. To maintain consistency and precision during the study period, quality control measures were implemented. All study personnel underwent training on the appropriate use of the InBody 270 to minimize error and ensure standardized procedures across all participants.

### 2.4 Statistical analysis

To analyze the data, achieve the research objectives, and test the hypotheses of the current study, IBM SPSS Statistics version 28 was utilized. Descriptive statistics in the form of frequencies and percentages for categorical variables and medians (interquartile range [IQR]) for numerically abnormally distributed variables were recorded. Pearson’s chi-squared test was applied to investigate the possible association and/or difference between two categorical variables, while the Mann–Whitney test was used to compare numerically abnormally distributed variables between two different groups. The effect size for Mann–Whitney test was computed using rank-biserial correlations coefficient. Binary and multivariate regression analyses were performed, and their results were expressed as crude and adjusted odds ratios by adding the low adherence to moderate adherence groups and testing them against high adherence groups to identify factors associated with high SHPDG adherence before and after controlling for confounders. *p* values of <0.05 were considered to indicate statistical significance.

## 3 Results

### 3.1 Characteristics of study participants

Table 1 presents the characteristics of the study sample. A total of 268 participants were included in the study (129 males [48.1%] and 139 females [51.9%]). Eighty-one participants were 18–24 years old (30.2%), 86 participants were 25–30 years old (32.1%), and 101 participants were 31–40 years old (37.7%). The age distribution differed significantly between males and females (*p* = 0.039), with more males in the 31–40 age group (43.4%) and more females in the 25–30 age group (38.8%). Most participants had a university education or higher (213, 79.5%) and were unmarried (179, 66.8%), with no statistically significant difference between sexes. Eleven participants were underweight (4.1%), 89 had normal weight (33.2%), 92 were overweight (34.3%), and 76 were obese (28.4%), with no significant sex differences.

**Table 1:** Characteristics of the study participants (*n* = 268).

Variable	Total *n* = 268		Male *n* = 129		Female *n* = 139		*p* value (male vs. female)*
	*n*	%	*n*	%	*n*	%	
Age (years)							
18–24	81	30.2	41	31.8	40	28.8	0.039
25–30	86	32.1	32	24.8	54	38.8	
31–40	101	37.7	56	43.4	45	32.4	
Education							
University or higher	213	79.5	103	79.8	110	79.1	0.886
High school or below	55	20.5	26	20.2	29	20.9	
Marital status							
Married	89	33.2	50	38.8	39	28.1	0.063
Unmarried	179	66.8	79	61.2	100	71.9	
BMI							
Underweight	11	4.1	5	3.9	6	4.3	0.354
Normal weight	89	33.2	36	27.9	53	38.1	
Overweight	92	34.3	52	40.2	40	28.8	
Obese I	52	19.4	26	20.2	26	18.7	
Obese II	16	6.0	6	4.7	10	7.2	
Obese III	8	3.0	4	3.1	4	2.9	
Body composition							
*Body fat mass*							
High	190	70.9	71	55.1	119	85.6	<0.001
Normal	60	22.4	43	33.3	17	12.2	
Low	18	6.7	15	11.6	3	2.2	
*Muscle mass*							
High	80	29.9	64	49.6	16	11.5	<0.001
Normal	139	51.8	59	45.7	80	57.6	
Low	49	18.3	6	4.7	43	30.9	
*Total body water*							
High	58	21.6	41	31.8	17	12.2	<0.001
Normal	170	63.5	82	63.5	88	63.3	
Low	40	14.9	6	4.7	34	24.5	

*Pearson’s chi-squared test.

There were significant differences between sexes across all body composition components (*p* < 0.001). Body fat mass was high in 190 participants (70.9%), with notably higher body fat mass in females (119, 85.6%) compared with males (71, 55.1%). However, more males had normal and low body fat mass (33.3 and 11.6%, respectively) than females (12.2 and 2.2%, respectively). Approximately half of the study participants had normal muscle mass (51.8%), with a greater proportion of males showing high muscle mass (49.6%) compared with females (11.5%). Conversely, low muscle mass was more common among females (30.9%) compared with males (4.7%). Total body water was within the normal range for most participants (63.5%); however, high total body water was more common in males (31.8%) than in females (12.2%).

### 3.2 SHPDG adherence

As shown in Table 2, most participants displayed moderate SHPDGs adherence (74.3%), with 13.1% demonstrating high SHPDGs adherence and 12.7% showing low SHPDGs adherence. Significantly more male participants showed high adherence to the SHPDGs compared with females (20.2% vs. 6.5%, *p* = 0.001). Fruits showed the lowest adherence, with 85.4% of participants never consuming them or only consuming 1–2 servings/day. Similarly, vegetables showed the low adherence, with 57.1% of participants never consuming them or only consuming 1–2 servings/day. Grain and bread intake showed a mixed pattern, with 51.5% of participants consuming 3–4 servings/day and 46.5% consuming only 1–2 servings/day. Milk and dairy products displayed low adherence, with 59.7% of participants never consuming them or only consuming one serving/day and only 12.7% meeting the recommended ≥3 servings/day. Meat and meat substitutes showed the highest adherence, with 58.2% of participants consuming ≥3 servings/day. Fat consumption was acceptable, with 46.6% of participants consuming minimal amounts and 47.4% consuming an appropriate amount daily. Only 22.4% of participants never consumed fast food and 61.9% consumed it 1–3 times/week. Only 38.1% of participants never consumed soft drinks and 30.2% consumed them ≥3 times/week. Crackers and crisps showed poor adherence, with 41.1% of participants consuming them 2–3 times/week or ≥4 times/week. Finally, only 23.5% of participants never consumed sweets or desserts, while 60.5% reported consuming them 1–3 times/week and 16.0% reported consuming them ≥4 times/week.

**Table 2:** SHPDG adherence levels of the study participants (*n* = 268).

Food group	Amount/day	Total *n* = 268 *n* (%)	Male *n* = 129 *n* (%)	Female *n* = 139 *n* (%)	*p* value*
1. Fruits/per day (e.g., Fresh, Dried, Juice, Cooked)	Never or 1–2 servings/day 3–4 servings/day ≥5 servings/day	229 (85.4) 31 (11.6) 8 (3.0)	105 (81.4) 20 (15.5) 4 (3.1)	124 (89.2) 11 (7.9) 4 (2.9)	0.148
2. Vegetables (e.g., fresh, dried, juice, cooked)	Never or 1–2 servings/day 3–4 servings/day ≥5 servings/day	153 (57.1) 78 (29.1) 37 (13.8)	72 (55.8) 40 (31.0) 17 (13.2)	81 (58.3) 38 (27.3) 20 (14.4)	0.798
3. Grains and bread (e.g., bread, toast, sandwich, rice, pasta, oats, corn flakes)	Never 1–2 servings/day 3–4 servings/day	5 (1.9) 125 (46.5) 138 (51.5)	2 (1.6) 35 (27.1) 92 (71.3)	3 (2.2) 90 (64.7) 46 (33.1)	<0.001
4. Milk and dairy products (e.g., milk, laban, yogurt, cheese, labneh, cream)	Never or 1 serving/day 2 servings/day ≥3 servings/day	160 (59.7) 74 (27.6) 34 (12.7)	62 (48.0) 42 (32.6) 25 (19.4)	98 (70.5) 32 (23.0) 9 (6.5)	<0.001
5. Meat and meat substitutes /per day (e.g., Sheep, Fish, Chicken, Egg, Legumes)	Never or 1 serving/day 2 servings/day ≥3 servings/day	45 (16.8) 67 (25.0) 156 (58.2)	10 (7.8) 22 (17.1) 97 (75.1)	35 (25.2) 45 (32.4) 59 (42.4)	<0.001
6. Fats (e.g., butter, ghee, oil)	Very little Some/appropriate amount A lot	125 (46.6) 127 (47.4) 16 (6.0)	62 (48.0) 57 (44.2) 10 (7.8)	63 (45.3) 70 (50.4) 6 (4.3)	0.374
7. Fast food (e.g., burger, pizza)	Never 1–3 times/week ≥4 times/week	60 (22.4) 66 (61.9) 42 (15.7)	37 (28.7) 73 (56.6) 19 (14.7)	23 (16.5) 93 (67.0) 23 (16.5)	0.058
8. Soft drinks	Never 1–2 times/week ≥3 times/week	102 (38.1) 85 (31.7) 81 (30.2)	40 (31.0) 38 (29.5) 51 (39.5)	62 (44.6) 47 (33.8) 30 (21.6)	0.005
9. Crackers and crisps/per week (e.g., Chips, Biscuit, Pretzel)	Never 1 time/week 2–3 times/week or ≥4 times/week	70 (26.1) 88 (32.8) 110 (41.1)	51 (39.5) 41 (31.8) 37 (28.7)	19 (13.7) 47 (33.8) 73 (52.5)	<0.001
10. Sweets and desserts (e.g., chocolate, donuts, basbousa, candy)	Never 1–3 times/week ≥4 times/week	63 (23.5) 162 (60.5) 43 (16.0)	44 (34.1) 76 (58.9) 9 (7.0)	19 (13.7) 86 (61.8) 34 (24.5)	<0.001
Overall SHPDG adherence	Low adherence	34 (12.7)	10 (7.8)	24(17.3)	
	Moderate adherence	199 (74.3)	93 (72.1)	106 (76.3)	0.001
	High adherence	35 (13.1)	26 (20.2)	9 (6.5)	

*Pearson’s chi-squared test.

There were no significant differences between the sexes in terms of fruit consumption (*p* = 0.148), vegetable consumption (*p* = 0.798), fat intake (*p* = 0.374), or fast food intake (*p* = 0.058). However, there were significant differences between males and females with respect to the intake of grains and bread, milk and dairy products, meat and meat substitutes, soft drinks, crackers and crisps, and sweets and desserts (*p* ≤ 0.005). Males reported higher intake of grains and bread at the 3–4 servings/day level (71.3%) compared with females (33.1%). Likewise, males reported higher consumption of meat and meat substitutes at the ≥3 servings/day level (75.1%) compared with females (42.4%). Furthermore, females reported lower intakes of milk and dairy products and soft drinks, with more female participants reporting never or minimal intake compared with males. In addition, females reported more frequent intake of crackers and crisps (52.5% at 2–3 times/week or ≥4 times/week among females vs. 28.7% among males). Finally, more males (34.1%) reported never consuming sweets and desserts compared with females (13.7%).

### 3.3 Physical activity

As shown in Table 3, the majority of participants demonstrated high physical activity (77.6%), while 16.8% were moderately active and only 5.6% reported low physical activity levels. There was a borderline significant sex differences (*p* = 0.068), with males reported higher levels of physical activity compared with females (83.7% vs. 71.9%). Mann–Whitney test revealed that males had significantly higher vigorous activity, moderate activity, walking, and total MET scores compared with females (*p* = 0.001, 0.001, 0.018, and <0.001, respectively), with effect sizes generally small (*r* = 0.207).

**Table 3:** Physical activity level and amount (MET min/week) of the study participants (*n* = 268).

	Total *n* = 268		Male *n* = 129		Female *n* = 139		*p* value*
	n	%	n	%	n	%	
Physical activity level							
Low (*n* = 15)	15	5.6	5	3.9	10	7.2	0.068
Moderate (*n* = 45)	45	16.8	16	12.4	29	20.9	
High (*n* = 208)	208	77.6	108	83.7	100	71.9	

	Median (IQR)	Median (IQR)	Mean rank	Median (IQR)	Mean rank	*p* value**
Amount of physical activity (MET min/week)						
Vigorous	2,160 (960–3,360)	2,400 (1360–3,840)	151.21	1,600 (720–2,880)	119	0.001
Moderate	600 (240–1,200)	840 (320–1,440)	151.08	480 (240–960)	119.12	0.001
Walking	495 (165–742.5)	594 (198–924)	146.07	346.5 (132–693)	123.76	0.018
Total METs	3,250 (1804–5,166)	4,000 (2524–5,719)	155.26	2,640 (1506–4,194)	115.23	<0.001

IQR, Interquartile range; SD, Standard deviation; *Pearson Chi-square test; **Mann–Whitney test.

### 3.4 Muscular endurance levels

As shown in Table 4, the majority of participants demonstrated below optimal endurance in the push-up tests, with 20.9% classified as having poor endurance, 19.4% as having below average endurance, and 25.8% as having average endurance. Only a minority achieved above optimal endurance levels, with 13.4% classified as having above average endurance, 7.1% as having good endurance, and 13.4% as having excellent endurance. Overall, push-up test performance differed significantly between males and females (*p* < 0.001). Females demonstrated a higher prevalence of poor muscular endurance compared with males (30.2% vs. 10.9%), while males demonstrated a higher prevalence of excellent muscular endurance compared with females (26.3% vs. 1.4%).

**Table 4:** Upper-body endurance levels of the study participants (*n* = 268).

Endurance level	Total *n* = 268		Male *n* = 129		Female *n* = 139		*p* value (male vs. female)
	*n*	%	*n*	%	*n*	%	
Poor	56	20.9	14	10.9	42	30.2	<0.001
Below average	52	19.4	23	17.8	29	20.9	
Average	69	25.8	21	16.3	48	34.6	
Above average	36	13.4	25	19.4	11	7.9	
Good	19	7.1	12	9.3	7	5.0	
Excellent	36	13.4	34	26.3	2	1.4	
Min	0		1		0		
Max	110		110		50		
Median (IQR)	15 (9–30)		30 (12–50)		10 (5–15)		
Mean (±SD)	20.96 (±19.46)		30.78 (±22.56)		11.85 (±9.30)		

### 3.5 Factors associated with SHPDG adherence

As shown in Table 5, the majority of participants displayed moderate adherence to the SHPDGs, with more males having high adherence than females (20.2% vs. 6.5%, *p* = 0.001). High SHPDG adherence was more common among participants with a high level of physical activity than among those with low or moderate levels (16.8% vs. 0.0%, *p* < 0.001). In addition, high adherence was more prevalent among participants with low fat mass than among those with high fat mass (33.3% vs. 8.4%, *p* = 0.007) and among those with high muscle mass than among those with low muscle mass (26.3% vs. 6.1%, *p* < 0.001). Similarly, participants with high total body water exhibited a greater proportion of high adherence than those with low total body water (19.0% vs. 7.5%, *p* = 0.022). Furthermore, a significant association was observed between muscular endurance and SHPDG adherence (*p* = 0.029). High SHPDG adherence increased across the muscular endurance categories, from 7.1% among participants with poor endurance to 30.5% among participants with excellent endurance, while low SHPDG adherence was most shown among participants with below average endurance (19.2%) and least common among participants with excellent endurance (2.8%). SHPDG adherence was not significantly associated with participant age, education level, marital status, or BMI.

**Table 5:** Factors associated with SHPDG adherence based on bivariate analysis.

Variable	Low SHPDG adherence *n* = 34 *n* (%)	Moderate SHPDG adherence *n* = 199 *n* (%)	High SHPDG adherence *n* = 35 *n* (%)	*p* value*
Sex				
Female (*n* = 139)	24 (17.3)	106 (76.3)	9 (6.5)	**0.001**
Male (*n* = 129)	10 (7.8)	93 (72.1)	26 (20.2)	
Age (years)				
18–24 (*n* = 81)	16 (19.8)	55 (67.9)	10 (12.3)	0.097
25–30 (*n* = 86)	10 (11.6)	68 (79.1)	8 (9.3)	
31–40 (*n* = 101)	8 (7.9)	76 (75.3)	17 (16.8)	
Education				
High school or below (*n* = 55)	10 (18.2)	37 (67.3)	8 (14.5)	0.330
University or higher (*n* = 213)	24 (11.3)	162 (76.1)	27 (12.7)	
Marital status				
Married (*n* = 89)	11 (12.4)	64 (71.9)	14 (15.7)	0.658
Unmarried (*n* = 179)	23 (12.8)	135 (75.4)	81 (11.7)	
BMI				
Underweight (*n* = 11)	3 (27.3)	8 (72.7)	0 (0.0)	0.230
Normal weight (*n* = 89)	13 (14.6)	63 (70.8)	13 (14.6)	
Overweight (*n* = 92)	12 (13.0)	69 (75.0)	11 (12.0)	
Obese I (*n* = 52)	4 (7.7)	37 (71.1)	11 (21.2)	
Obese II (*n* = 16)	2 (12.5)	14 (87.5)	0 (0.0)	
Obese III (*n* = 8)	0 (0.0)	8 (100)	0 (0.0)	
Physical activity level				
Low (*n* = 15)	5 (33.3)	10 (66.7)	0 (0.0)	**<0.001**
Moderate (*n* = 45)	11 (24.4)	34 (75.6)	0 (0.0)	
High (*n* = 208)	18 (8.7)	155 (74.5)	35 (16.8)	
Fat mass				
Low (*n* = 18)	2 (11.1)	10 (55.6)	6 (33.3)	**0.007**
Normal (*n* = 60)	6 (10.0)	41 (68.3)	13 (21.7)	
High (*n* = 190)	26 (13.7)	148 (77.9)	16 (8.4)	
Muscle mass				
Low (*n* = 49)	12 (24.5)	34 (69.4)	3 (6.1)	**<0.001**
Normal (*n* = 139)	18 (12.9)	110 (79.2)	11 (7.9)	
High (*n* = 80)	4 (5.0)	55 (68.7)	21 (26.3)	
Total body water				
Low (*n* = 40)	11 (27.05)	26 (65.0)	3 (7.5)	**0.022**
Normal (*n* = 170)	18 (10.6)	131 (77.1)	21 (12.4)	
High (*n* = 58)	5 (8.6)	42 (72.4)	11 (19.0)	
Push-up test				
Poor (*n* = 56)	10 (17.9)	42 (75.0)	4 (7.1)	**0.029**
Below average (*n* = 52)	10 (19.2)	38 (73.1)	4 (7.7)	
Average (*n* = 69)	7 (10.1)	55 (79.8)	7 (10.1)	
Above average (*n* = 36)	5 (13.9)	26 (72.2)	5 (13.9)	
Good (*n* = 19)	1 (5.3)	14 (73.6)	4 (21.1)	
Excellent (*n* = 36)	1 (2.8)	24 (66.7)	11 (30.5)	

*Pearson’s chi-squared test. Bold values indicate statistically significant *p*-values (*p* < 0.05).

As shown in Table 6, multivariate logistic regression analysis revealed that, after controlling for potential confounding factors, only participants with high muscle mass were significantly more likely to have a high level of SHPDG adherence compared with their counterparts (adjusted odds ratio [aOR] = 4.99, 95% confidence interval [CI] = 1.43–17.39, *p* = 0.008). By contrast, participants with high fat mass were significantly less likely to have a high level of SHPDG adherence (aOR = 0.17, 95% CI = 0.03–0.97, *p* = 0.046). Male participants and those with excellent push-up test performance were found to be more likely than females and those with poor push-up performance to have high SHPDG adherence in the binary analysis; however, these associations were no longer significant after adjustment in the multivariate analysis.

**Table 6:** Factors associated with high SHPDG adherence.

Independent variable	SHPDG adherence		cOR (95% CI)	*p* value	aOR (95% CI)	*p* value
	Low/moderate *n* = 233 *n* (%)	High *n* = 35 *n* (%)				
Sex						
Female (*n* = 139)	130 (93.5)	9 (6.5)	1.0		1.0	
Male (*n* = 129)	103 (79.8)	26 (20.2)	3.65 (1.64–8.12)	0.001	0.75 (0.22–2.53)	0.646
Age (years)						
18–24 (*n* = 81)	71 (87.7)	10 (12.3)	1.0		1.0	
25–30 (*n* = 86)	78 (90.7)	8 (9.3)	0.73 (0.27–1.95)	0.527	0.52 (0.15–1.74)	0.285
31–40 (*n* = 101)	84 (83.2)	17 (16.8)	1.44 (0.62–3.34)	0.399	1.08 (0.31–3.81)	0.904
Education						
High school or below (*n* = 55)	47 (85.5)	8 (14.5)	1.0		1.0	
University or higher (*n* = 213)	186 (87.3)	27 (12.7)	0.85 (0.36–2.00)	0.714	0.75 (0.26–2.20)	0.601
Marital status						
Unmarried (*n* = 179)	158 (88.3)	21 (11.7)	1.0		1.0	
Married (*n* = 89)	75 (84.3)	14 (15.7)	1.40 (0.68–2.91)	0.360	1.21 (0.43–3.44)	0.722
BMI						
Underweight (*n* = 11)	11 (100)	0 (0.0)	1.0		1.0	
Normal weight (*n* = 89)	76 (85.4)	13 (14.6)	1.88 (0.22–15.83)	0.561	1.79 (0.26–14.02)	0.611
Overweight (*n* = 92)	81 (88.0)	11 (12.0)	1.49 (0.18–12.72)	0.713	1.41 (0.16–13.12)	0.715
Obese I (*n* = 52)	41 (78.8)	11 (21.2)	2.95 (0.34–25.40)	0.324	2.77 (0.31–20.25)	0.310
Obese II (*n* = 16)	16 (100)	0 (0.0)	0.69 (0.04–12.20)	0.799	0.81 (0.03–11.99)	0.807
Obese III (*n* = 8)	8 (100)	0 (0.0)	1.38 (0.07–25.43)	0.831	1.32 (0.09–26.03)	0.926
Physical activity level						
Low (*n* = 15)	15 (100)	0 (0.0)	1.0		1.0	
Moderate (*n* = 45)	45 (100)	0 (0.0)	0.33 (0.02–5.66)	0.447	0.49 (0.03–6.08)	0.450
High (*n* = 208)	173 (83.2)	35 (16.8)	3.03 (0.39–23.73)	0.290	3.23 (0.32–20.58)	0.196
Fat mass						
Low (*n* = 18)	12 (66.7)	6 (33.3)	1.0		1.0	
Normal (*n* = 60)	47 (78.3)	13 (21.7)	0.55 (0.17–1.76)	0.316	0.47 (0.10–2.23)	0.343
High (*n* = 190)	174 (91.6)	16 (8.4)	0.18 (0.06–0.56)	0.003	0.17 (0.03–0.97)	0.046
Muscle mass						
Low (*n* = 49)	46 (93.9)	3 (6.1)	1.0		1.0	
Normal (*n* = 139)	128 (92.1)	11 (7.9)	1.32 (0.35–4.93)	0.682	1.30 (0.39–5.01)	0.741
High (*n* = 80)	59 (73.8)	21 (26.3)	5.46 (1.53–19.43)	0.009	4.99 (1.43–17.39)	0.008
Total body water						
Low (*n* = 40)	37 (92.5)	3 (7.5)	1.0		1.0	
Normal (*n* = 170)	149 (87.6)	21 (12.4)	1.74 (0.49–6.14)	0.391	1.70 (0.52–6.01)	0.392
High (*n* = 58)	47 (81.0)	11 (19.0)	2.89 (0.75–11.11)	0.123	1.89 (0.68–10.28)	0.201
Push-up test						
Poor (*n* = 56)	52 (92.9)	4 (7.1)	1.0		1.0	
Below average (*n* = 52)	48 (92.3)	4 (7.7)	1.08 (0.26–4.57)	0.913	0.55 (0.10–3.18)	0.504
Average (*n* = 69)	62 (89.9)	7 (10.1)	1.47 (0.41–5.29)	0.558	0.81 (0.18–3.74)	0.791
Above average (*n* = 36)	31 (86.1)	5 (13.6)	2.10 (0.52–8.40)	0.296	0.70 (0.12–3.94)	0.685
Good (*n* = 19)	15 (78.9)	4 (21.1)	3.47 (0.77–15.54)	0.104	1.34 (0.19–9.24)	0.766
Excellent (*n* = 36)	25 (69.4)	11 (30.6)	5.72 (1.66–19.76)	0.006	0.96 (0.16–5.68)	0.960

cOR, Crude odds ratio aOR: Adjusted odds ratio.

## 4 Discussion

The aim of this study was to assess the associations between SHPDG adherence and physical activity level, muscular endurance, and body composition among male and female adult gym users in Saudi Arabia. The study findings may help to increase awareness about healthy dietary guidelines among gym users, who are more likely to adhere to various dietary restrictions and types of nutritional supplementation than the fundamental dietary guidelines. In addition, the results of this study could assist nutritionists, dietitians, and fitness professionals in establishing more sex-stratified, evidence-based dietary educational programs highlighting the importance of following the healthy dietary guidelines. Furthermore, given the well-established link between body composition and cardiometabolic disease risk, these findings reinforce that dietary quality remains an independent modifiable factor even among physically active individuals. This highlights the importance of integrating structured dietary guidance into fitness-based programs, rather than treating adherence to dietary guidelines as secondary to exercise. Finally, the obtained results may guide future studies relating to adherence to dietary guidelines.

Overall, the findings of this study revealed significant associations between SHPDG adherence and physical activity levels, muscular endurance, and body composition, with some sex differences observed across these associations. The results revealed an overall moderate adherence to the SHPDGs among 74.3% of participants, with 13.1% of participants reporting high adherence and 12.7% reporting low adherence. This pattern of moderate adherence may be attributable to consistently low consumption of key food groups central to the SHPDGs, particularly fruits, vegetables, and dairy. This aligns with the findings reported by Alahmadi and Albassam, who assessed nutritional knowledge and dietary intake among physically active practitioners and athletes in Riyadh and found that fruits, vegetables, and dairy were the lowest scoring dietary intake domains, while protein-rich foods such as meat, fish, and chicken were consumed at comparatively higher levels (35). Moreover, the widespread accessibility of unhealthy foods at low cost, inadequate food preparation skills, and a lack of motivation to eat healthily foods may prevent progression from moderate to high adherence (37).

Male participants were more likely to show high adherence to the SHPDGs than females (20.2% vs. 6.5%). However, this is not aligned with the majority of studies, which reported that females tend to display higher overall dietary guideline adherence than males (42). This could be related to the fact that previous studies were conducted among the general population, whereas the current study focused on gym users. In previous work, sporting performance goals were identified as key drivers of dietary change among physically active young men (43, 44). By contrast, women are more likely than men to report appearance and weight loss as their drivers of dietary behavior, which may explain why females demonstrate significantly higher levels of cognitive dietary restraint and emotional eating, patterns that are associated with less sustainable long-term dietary adherence (45, 46). The present findings highlight that physical activity setting and goal orientation are contextual factors that can invert typically observed sex differences in terms of adherence to dietary guidelines.

With respect to food groups, only 3.0% of respondents met the highest level of recommended fruit intake (≥5 servings/day) and 13.8% met the highest level of vegetable intake (≥5 servings/day), with no significant differences between males and females. These results indicate that the participants may not be consuming essential vitamins, minerals, and dietary fiber. Adequate intake of vitamins and minerals is particularly important for gym users because their involvement in energy-yielding metabolism, oxygen transport, and neuronal function makes them critical for muscular function, while physical activity coupled with insufficient micronutrient intake may lead to vitamin and mineral deficiencies that impair athletic performance and health (47, 48). Furthermore, dietary fiber improve gastrointestinal health, support energy release, and glycemic regulation, which are important for supporting training and improving body composition in gym users (49, 50). Future studies need to assess the intake patterns of certain micronutrient and dietary fiber of male and female gym users in relation to their training goals. This may supply insight into dietary gaps and facilitate planning for dietary education that meets the nutritional needs of active population.

Over half of participants stated consuming 3–4 servings/day of whole grains (51.5%), with pattern is more common among males (71.3%) compared to females (33.1%). This could reflect a tendency to prioritize high protein foods over carbohydrate-rich sources, even with the established role of carbohydrates in supported energy release and glycemic regulation during exercise. In contrast, the lower grain intake reported by females may be due to a tendency to restrict carbohydrate-rich foods, reflecting the popularity of low-carbohydrate and ketogenic dietary practices within fitness communities (51, 52). This is concerning given that evidence indicates that low-carbohydrate, high-fat diets may impair exercise economy and negate performance benefits from training (53). Thus, it is important to encourage gym users to meet their needs from whole grains to support their health goals of losing weight or improving fitness performance. Whole grains exert their health benefits via various mechanisms, including their high dietary fiber content, low glycemic index, and ability to stimulate the release of satiety hormones such as peptide YY and glucagon-like peptide-1, all of which contribute to reduced appetite and better energy regulation (54).

The majority of participants reported low SHPDG adherence with respect to the consumption of dairy products, with 59.7% consuming these items never or consuming only one serving/day. About one-quarter of participants reported moderate adherence by consuming two servings/day (27.6%), while 12.7% reported high adherence by consuming ≥3 servings/day. In addition, there was a sex difference, with high consumption more prevalent among males than females (19.4% vs. 6.5%). This finding was unexpected because dairy products are an important food group for physically active people to meet their nutritional needs, support muscle protein synthesis, and improve post-exercise recovery, and they also contain calcium and vitamin D, which are fundamental to maintaining bone density and musculoskeletal health (21). Future studies need to examine low intake of dairy products among gym populations to better understand the underlying determinants in this specific context. Furthermore, in the present study we did not assess physiological intolerance, supplement substitution intake, and fitness culture misconceptions. Therefore, the results should be interpreted with caution.

Most of the study participants showed high SHPDG adherence with respect to meat and meat substitutes, with 58.2% consuming ≥3 servings/day and 25.0% falling within the moderate consumption category of two servings/day. Only 16.8% showed low adherence by never consuming these food items or only consuming one serving/day. In regard to sex differences, the high intake was more predominant among male participants than among female participants (75.1% vs. 42.4%). The high intake of meat and meat substitutes seen among gym users is reflecting the emphasis on protein consumption within fitness and gym culture. Protein-rich foods, including meat, poultry, fish, eggs, and legumes, are familiar among physically active individuals as important for muscle protein synthesis, strength development, and post-exercise recovery (21). However, this positive result needs to be interpreted cautiously within the dietary context of this study as the high consumption of protein-rich foods linked with low intake of other food groups such as fruits, dairy, and vegetables. This may suggest a pattern of selective dietary adherence that prioritizes protein intake over overall dietary balance. This selective dietary pattern could lead to micronutrient deficiencies and negative long-term health outcomes, highlighting the need for comprehensive adherence to the SHPDGs among gym users rather than the prioritization of a single food group.

In regard to fat consumption, most of the study participants achieved either high SHPDG adherence by consuming minimal amounts of fat daily (46.6%) or moderate adherence by consuming an appropriate amount (47.4%), while only a small proportion reported low-adherence category of a high fat intake (6.0%). There were no significant differences between males and females. This result may reflect a general awareness among gym users of the influence of excessive fat consumption on body composition and physical performance.

In terms of fast food consumption, only 22.4% of participants reported high SHPDG adherence by never consuming fast food, while most of the participants fell within the moderate-adherence category of 1–3 times/week (61.9%). The low adherence was showed with a notable percentage, who reported fast food consumption of ≥4 times/week (15.7%). These results indicate that fast food consumption remains prevalent among gym users. The regular consumption of fast food, which is characterized by high saturated fat, refined carbohydrates, and high sodium content, might directly challenge the body composition goals of gym users and counteract the benefits of training (55). This pattern could be due to the influence of westernized dietary culture among Saudi adults and may explain the elevated rates of overweight and obesity observed in this study (56). The soft drinks consumption also exposed a concerning adherence pattern, with only 38.1% of participants reported high adherence by never consuming soft drinks, while nearly 61.9% stated regular consumption (1–2 times/week or ≥3 times/week). Significant sex differences were detected (*p* = 0.005), with a higher proportion of males than females showing the highest level of soft drink consumption of ≥3 times/week (39.5% vs. 21.6%) and a higher proportion of females than males reporting never consuming soft drinks (44.6% vs. 31.0%). This higher intake among males may reflect differing dietary attitudes between sexes, with males being less conscious of their beverage choices. Regular sugary beverage intake in a gym users is concerning given the well-established relationship between sugary beverages and excess caloric intake, insulin resistance, and increased body fat accumulation, contributing to the higher rates of overweight and obesity observed in this study sample.

With respect to crackers and crisps consumption, nearly three-quarters of participants stated regular consumption of (1 time/week, 2–3 times/week, or ≥4 times/week; 73.9%). Females displayed lower SHPDG adherence, defined as consuming crackers and crisps 2–3 times/week or ≥4 times/week, than males (52.5% vs. 28.7%). This pattern could possibly attributable to snacking behaviors driven by the emotional eating and dietary restraint cycles generally observed among young women (57). The association between processed snack consumption and excess caloric intake, poor dietary quality, and increased body fat accumulation could explain the higher body fat mass observed among females in this study. In addition, sweet and dessert intake showed poor adherence, with females reported higher low SHPDG adherence than males (24.5% vs. 7.0%), driven by emotional eating behaviors. This, combined with higher processed snack intake, contributes to the higher body fat percentage seen among females, indicating the need for sex-specific dietary interventions targeting emotional eating among female gym users.

The physical activity results revealed that the majority of participants reported a high physical activity level (77.6%), while 16.8% were moderately active and only 5.6% reported a low physical activity level. No significant sex differences were observed in terms of total physical activity level (*p* = 0.068); however, males had higher vigorous activity, moderate activity, walking, and total MET scores compared with females (*p* = 0.001, *p* = 0.001, *p* = 0.018, and *p* < 0.001, respectively). SHPDG adherence among the participants was associated with the level of physical activity, which reflects health-aware behavioral patterns among the study sample. A balanced and healthy diet could enhance physical activity level via various biological mechanisms. Based on a recent review, carbohydrates are the predominant source of energy for moderate-to-intense exercise and their deficiency leads to glycogen depletion, which has a major impact on performance (58). Whether during training or competition, sufficient carbohydrate intake before and during exercise is recommended based on the exercise type and duration (53). Furthermore, consuming adequate quantities of micronutrients, including folate, vitamin B_12_, iron, and magnesium, helps prevent declines in oxygen transport and physical activity performance (54). Research shows that both males and females who follow healthier eating habits tend to engage in higher levels of moderate-to-vigorous physical activity and maintain a state of metabolic health, especially when they consume more fruits, vegetables, and fish (55–57).

The significant association between muscular endurance and SHPDG adherence, with high adherence increasing from 7.1% among those with poor endurance to 30.5% among those with excellent endurance, highlights the role of balanced dietary guideline adherence in improving muscular performance. Studies show that adherence to balanced dietary guidelines, primarily an adequate intake of complex carbohydrates, high-quality protein, calcium, and vitamin D, is essential to sustaining muscular endurance and physical performance (21). Beyond dietary adherence, significant sex differences in terms of muscular endurance were also observed (*p* < 0.001), with males displaying greater endurance than females (excellent endurance: 26.3% vs. 1.4%). This is consistent with well-documented physiological advantages in males, including greater testosterone-driven muscle protein synthesis and higher lean muscle mass (59), and it was further reflected in the binary logistic regression analysis results, which confirmed that males were significantly more likely to achieve higher upper-body muscular endurance scores than females — consistent with established evidence that males typically outperform females in push-up and bench-press exercises owing to greater upper-body strength (60).

The poorer muscular endurance observed among females may partly reflect their nutritionally imbalanced dietary patterns, characterized by insufficient dairy and whole grain intake alongside higher consumption of fast food, processed snacks, and sweets, which may compromise the nutritional requirements for muscle function and endurance capacity. Inadequate calcium and vitamin D intake from dairy products has been linked to impaired neuromuscular function and reduced muscular endurance (61), while lower whole-grain consumption may deprive muscles of the sustained glycemic support necessary for high endurance (21). Furthermore, excessive consumption of energy-dense, nutrient-poor foods has been associated with systemic inflammation and compromised physical performance (62), suggesting that the dietary imbalances observed among female gym users in the present study may represent a meaningful nutritional barrier to achieving optimal muscular endurance outcomes.

The significant associations between SHPDG adherence and all body composition components reinforce the significant role of balanced dietary guideline adherence in determining body composition. The inverse relationship between dietary adherence and fat mass aligns with established evidence that greater adherence to dietary patterns high in fruits, vegetables, whole grains, and legumes is associated with more favorable outcomes related to body weight and reduced risk of obesity. The poor dietary patterns observed in this study, characterized by low fruit, dairy, and whole-grain intake combined with excessive intake of fast food, and processed snacks, likely causes the high prevalence of elevated fat mass, particularly among females, which is consistent with evidence that dietary patterns lower in fruits, vegetables, and whole grains and high in added sugars and refined grains are connected with unfavorable body weight and obesity outcomes.

The positive relationship between SHPDG adherence and muscle mass is consistent with evidence that adequate dietary protein intake is crucial for stimulating muscle protein synthesis, a metabolic process in which ingested amino acids are synthesized into muscle proteins, thereby preserving lean mass and optimizing body composition (63). After adjusting for potential confounders using multivariate analysis, the significant associations between dietary guideline adherence and both muscle mass and fat mass confirm that dietary intake has a direct physiological effect on adiposity and lean tissue regulation.

The association between SHPDG adherence and total body water additionally reflects the physiological link between lean muscle mass and hydration status, considering that skeletal muscle is among the most hydrated tissues in the human body with a water content of approximately 75% by mass, where even a modest decrease in total body water on the order of 3–4% can significantly impair muscle strength, power, and endurance (64). These findings collectively suggest that higher dietary adherence supports greater lean mass, which in turn sustains optimal body water distribution in physically active individuals.

This study observed sex differences in body composition which was also consistent with previous physiological evidence. Sex steroid hormones are the primary driver of body composition dimorphism during pubertal growth, with males exhibiting higher skeletal muscle mass, especially in the upper body, contributing to greater strength, whereas females have inherently higher fat mass affected by hormonal factors (65). In addition, these differences are compounded by dietary behavior, as a cross-sectional study conducted in Makkah indicated that females displayed unhealthy dietary patterns characterized by high sugar and carbohydrate consumption (66). Likewise, poor compliance with Saudi dietary guidelines was reported among female university students (37), supporting the need for sex-specific dietary interventions among gym users.

This study has several strengths, including a near-equal sex representation that allowed for sex-based comparisons, the use of body composition measurements to enable a multidimensional evaluation beyond BMI assessment, and the application of the SHPDG adherence scoring approach as a culturally relevant dietary assessment tool specific to the Saudi population. However, several limitations must be acknowledged. The cross-sectional design prevents the establishment of causal relationships between dietary guideline adherence and the observed outcomes, and future studies are warranted to confirm the observed associations. Dietary intake was evaluated through self-reporting, which is susceptible to recall and social desirability bias, while recruitment mainly from gyms within a specific region introduces selection bias, limiting the generalizability of the study findings. In addition, the absence of data on nutritional supplementation and food intolerance is a significant limitation given their potential confounding influences on dietary intake and practices. The SHPDG adherence scoring approach does not capture total caloric intake, which may be an important confounding factor in interpreting dietary guideline adherence and body composition relationships.

## 5 Conclusion

This study shows that gym attendees in Saudi Arabia demonstrate moderate adherence to the SHPDGs, with critical inadequacies in fruit, vegetable, and dairy intake alongside with high intake of fast food, processed snacks, and sweets. In addition, sex differences in terms of dietary adherence were paralleled by significant disparities in body composition and muscular endurance, with females showing higher body fat mass and inferior muscular endurance despite regular gym attendance. These findings challenge the assumption that gym attendance reflects healthy dietary practices and provide compelling evidence that moderate dietary guideline adherence is insufficient to optimize body composition and muscular endurance outcomes. Sex-specific, evidence-based nutritional counseling integrated within gym settings is imperative to redirect physically active individuals toward comprehensive dietary guideline adherence as the fundamental foundation for achieving sustainable body composition and meeting performance goals.

## Data Availability

The original contributions presented in the study are included in the article, further inquiries can be directed to the corresponding authors.
