# Supplementary material for: Association between adherence to Saudi healthy plate dietary guidelines and physical activity, muscular endurance, and body composition among adult gym users
Source: Front Nutr. 2026 Sep 16;13:1940147. doi: 10.3389/fnut.2026.1940147 (PMC13623615; doi:10.3389/fnut.2026.1940147)
Supplement: Supplementary file 1 [file Table_1.docx]

Supplementary Material

# 1 Supplementary Tables

**Table S1:** The Saudi Healthy Plate Dietary Guidelines (SHPDGs) Scores.

| **Food Groups** | **Options** | **Score** |
| --- | --- | --- |
| 1. Fruits/per day (e.g., Fresh, Dried, Juice, Cooked) | ≥5 Serving | 0 |
|  | 3–4 Serving | 1 |
|  | 1–2 Serving | 2 |
|  | Never | 2 |
| 2. Vegetables/per day (e.g., Fresh, Dried, Juice, Cooked) | ≥5 Serving | 0 |
|  | 3–4 Serving | 1 |
|  | 1–2 Serving | 2 |
|  | Never | 2 |
| 3. Grains & Bread /per day (e.g., Bread, Toast, Sandwich, Rice, Pasta, Oats, Corn flacks) | 3–4 Serving | 0 |
|  | 1–2 Serving | 1 |
|  | Never | 2 |
| 4. Milk & Dairy Products /per day (e.g., Milk, Laban, Yogurt, Cheses, Labneh, Cream) | ≥3 Serving | 0 |
|  | 2 Serving | 1 |
|  | 1 Serving | 2 |
|  | Never | 2 |
| 5. Meat & Meat Substitutes /per day (e.g., Sheep, Fish, Chicken, Egg, Legumes) | ≥3 Serving | 0 |
|  | 2 Serving | 1 |
|  | 1 Serving | 2 |
|  | Never | 2 |
| 6. Fats /per day (e.g., Butter, Ghee, Oil) | Very little | 0 |
|  | Some/Appropriate amount | 1 |
|  | A lot | 2 |
| 7. Fast Foods /per week (e.g., Burger, Pizza) | Never | 0 |
|  | 1–3 Times | 1 |
|  | ≥4 Times | 2 |
| 8. Soft Drinks /per week | Never | 0 |
|  | 1–2 Times | 1 |
|  | ≥3 Times | 2 |
| 9. Crackers & Crisps /per week (e.g., Chips, Biscuit, Pretzel) | Never | 0 |
|  | 1 Time | 1 |
|  | 2–3 Times | 2 |
|  | ≥4 Times | 2 |
| 10. Sweet & Dessert /per week (e.g., Chocolate, Donut, Basbousa, Candy) | Never | 0 |
|  | 1–3 Times | 1 |
|  | ≥4 Times | 2 |

**Table S2:** Normative Data for Push-Ups for Males and Females

| **Male** | | | | | | |
| --- | --- | --- | --- | --- | --- | --- |
| **Age** | **Excellent** | **Good** | **Above Average** | **Average** | **Below Average** | **Poor** |
| **17–19** | >56 | 47–56 | 35–46 | 19–34 | 11–18 | <11 |
| **20–29** | >47 | 39–47 | 30–38 | 17–29 | 10–16 | <10 |
| **30–39** | >41 | 34–41 | 25–33 | 13–24 | 8–12 | <8 |
| **40–49** | >34 | 28–34 | 21–27 | 11–20 | 6–10 | <6 |
| **50–59** | >31 | 25–31 | 18–24 | 9–17 | 5–8 | <5 |
| **60–65** | >30 | 24–30 | 17–23 | 6–16 | 3–5 | <3 |
| **Female** | | | | | | |
| **17–19** | >35 | 27–35 | 21–26 | 11–20 | 6–10 | <6 |
| **20–29** | >36 | 30–36 | 23–29 | 12–22 | 7–11 | <7 |
| **30–39** | >37 | 30–37 | 22–29 | 10–21 | 5–9 | <5 |
| **40–49** | >31 | 25–31 | 18–24 | 8–17 | 4–7 | <4 |
| **50–59** | >25 | 21–25 | 15–20 | 7–14 | 3–6 | <3 |
| **60–65** | >23 | 19–23 | 13–18 | 5–12 | 2–4 | <2 |

Data adopted from (Golding *et al*, 1986)
